# Preoperative breast MRI positively impacts surgical outcomes of needle biopsy–diagnosed pure DCIS: a patient-matched analysis from the MIPA study

**DOI:** 10.1007/s00330-023-10409-5

**Published:** 2023-11-24

**Authors:** Andrea Cozzi, Giovanni Di Leo, Nehmat Houssami, Fiona J. Gilbert, Thomas H. Helbich, Marina Álvarez Benito, Corinne Balleyguier, Massimo Bazzocchi, Peter Bult, Massimo Calabrese, Julia Camps Herrero, Francesco Cartia, Enrico Cassano, Paola Clauser, Marcos F. de Lima Docema, Catherine Depretto, Valeria Dominelli, Gábor Forrai, Rossano Girometti, Steven E. Harms, Sarah Hilborne, Raffaele Ienzi, Marc B. I. Lobbes, Claudio Losio, Ritse M. Mann, Stefania Montemezzi, Inge-Marie Obdeijn, Umit Aksoy Ozcan, Federica Pediconi, Katja Pinker, Heike Preibsch, José L. Raya Povedano, Carolina Rossi Saccarelli, Daniela Sacchetto, Gianfranco P. Scaperrotta, Margrethe Schlooz, Botond K. Szabó, Donna B. Taylor, Sila Ö. Ulus, Mireille Van Goethem, Jeroen Veltman, Stefanie Weigel, Evelyn Wenkel, Chiara Zuiani, Francesco Sardanelli

**Affiliations:** 1 Unit of Radiology, IRCCS Policlinico San Donato, Via Rodolfo Morandi 30, 20097 San Donato Milanese, Italy; 2 Present Address: Imaging Institute of Southern Switzerland, Ente Ospedaliero Cantonale, Lugano, Switzerland; 3 The Daffodil Centre, Faculty of Medicine and Health, The University of Sydney (Joint Venture with Cancer Council NSW), Sydney, Australia; 4 Department of Radiology, School of Clinical Medicine, Cambridge Biomedical Campus, University of Cambridge, Cambridge, UK; 5 Division of General and Paediatric Radiology, Department of Biomedical Imaging and Image-Guided Therapy, Medical University of Vienna, Vienna, Austria; 6 Division of Molecular and Structural Preclinical Imaging, Department of Biomedical Imaging and Image-Guided Therapy, Medical University of Vienna, Vienna, Austria; 7 Department of Radiology, Hospital Universitario Reina Sofía, Córdoba, Spain; 8 Department of Radiology, Institut Gustave Roussy, Villejuif, France; 9 Biomaps, UMR1281 INSERM, CEA, CNRS, Université Paris-Saclay, Villejuif, France; 10 Institute of Radiology, Department of Medicine, Ospedale Universitario S. Maria della Misericordia, Università degli Studi di Udine, Udine, Italy; 11 Department of Pathology, Radboud University Medical Center, Nijmegen, The Netherlands; 12 Unit of Oncological and Breast Radiology, IRCCS Ospedale Policlinico San Martino, Genoa, Italy; 13 Department of Radiology, Hospital Universitario de La Ribera, Alzira, Spain; 14 Present Address: Ribera Salud Hospitals, Valencia, Spain; 15 Unit of Breast Imaging, Fondazione IRCCS Istituto Nazionale dei Tumori, Milan, Italy; 16 Breast Imaging Division, IEO, European Institute of Oncology IRCCS, Milan, Italy; 17 Department of Radiology, Hospital Sírio Libanês, São Paulo, Brazil; 18 Department of Radiology, MHEK Teaching Hospital, Semmelweis University, Budapest, Hungary; 19 Present Address: Department of Radiology, Duna Medical Center, GE-RAD Kft, Budapest, Hungary; 20 Breast Center of Northwest Arkansas, Fayetteville, AR USA; 21 Department of Radiology, Di.Bi.MED, Policlinico Universitario Paolo Giaccone Università degli Studi di Palermo, Palermo, Italy; 22 Department of Radiology and Nuclear Medicine, Maastricht University Medical Center, Maastricht, The Netherlands; 23 Present Address: Department of Medical Imaging, Zuyderland Medical Center, Sittard-Geleen, The Netherlands; 24 Department of Breast Radiology, IRCCS Ospedale San Raffaele, Milan, Italy; 25 Department of Radiology and Nuclear Medicine, Radboud University Medical Center, Nijmegen, The Netherlands; 26 Department of Radiology, The Netherlands Cancer Institute, Amsterdam, The Netherlands; 27 Department of Radiology, Azienda Ospedaliera Universitaria Integrata Verona, Verona, Italy; 28 Department of Radiology and Nuclear Medicine, Erasmus University Medical Center, Rotterdam, The Netherlands; 29 Department of Radiology, Acıbadem Atasehir Hospital, Istanbul, Turkey; 30 Department of Radiological, Oncological and Pathological Sciences, Università degli Studi di Roma “La Sapienza”, Rome, Italy; 31 Department of Radiology, Breast Imaging Service, Memorial Sloan Kettering Cancer Center, New York, NY USA; 32 Department of Diagnostic and Interventional Radiology, University Hospital of Tübingen, Tübingen, Germany; 33 Kiwifarm S.R.L., La Morra, Italy; 34 Disaster Medicine Service 118, ASL CN1, Levaldigi, Italy; 35 Department of Surgery, Radboud University Medical Center, Nijmegen, The Netherlands; 36 Department of Radiology, Barking Havering and Redbridge University Hospitals NHS Trust, London, UK; 37 Medical School, Faculty of Health and Medical Sciences, The University of Western Australia, Perth, Australia; 38 Department of Radiology, Royal Perth Hospital, Perth, Australia; 39 Gynecological Oncology Unit, Department of Obstetrics and Gynecology, Department of Radiology, Multidisciplinary Breast Clinic, Antwerp University Hospital, University of Antwerp, Antwerp, Belgium; 40 Maatschap Radiologie Oost-Nederland, Oldenzaal, The Netherlands; 41 Clinic for Radiology and Reference Center for Mammography, University of Münster, Münster, Germany; 42 Department of Radiology, University Hospital of Erlangen, Erlangen, Germany; 43 Department of Biomedical Sciences for Health, Università degli Studi di Milano, Milan, Italy

**Keywords:** Breast neoplasms (biopsy, needle), Carcinoma (intraductal, noninfiltrating), Magnetic resonance imaging, Mastectomy, Reoperation

## Abstract

**Objectives:**

To investigate the influence of preoperative breast MRI on mastectomy and reoperation rates in patients with pure ductal carcinoma in situ (DCIS).

**Methods:**

The MIPA observational study database (7245 patients) was searched for patients aged 18–80 years with pure unilateral DCIS diagnosed at core needle or vacuum-assisted biopsy (CNB/VAB) and planned for primary surgery. Patients who underwent preoperative MRI (MRI group) were matched (1:1) to those who did not receive MRI (noMRI group) according to 8 confounding covariates that drive referral to MRI (age; hormonal status; familial risk; posterior-to-nipple diameter; BI-RADS category; lesion diameter; lesion presentation; surgical planning at conventional imaging). Surgical outcomes were compared between the matched groups with nonparametric statistics after calculating odds ratios (ORs).

**Results:**

Of 1005 women with pure unilateral DCIS at CNB/VAB (507 MRI group, 498 noMRI group), 309 remained in each group after matching. First-line mastectomy rate in the MRI group was 20.1% (62/309 patients, OR 2.03) compared to 11.0% in the noMRI group (34/309 patients, *p* = 0.003). The reoperation rate was 10.0% in the MRI group (31/309, OR for reoperation 0.40) and 22.0% in the noMRI group (68/309, *p* < 0.001), with a 2.53 OR of avoiding reoperation in the MRI group. The overall mastectomy rate was 23.3% in the MRI group (72/309, OR 1.40) and 17.8% in the noMRI group (55/309, *p* = 0.111).

**Conclusions:**

Compared to those going directly to surgery, patients with pure DCIS at CNB/VAB who underwent preoperative MRI had a higher OR for first-line mastectomy but a substantially lower OR for reoperation.

**Clinical relevance statement:**

When confounding factors behind MRI referral are accounted for in the comparison of patients with CNB/VAB-diagnosed pure unilateral DCIS, preoperative MRI yields a reduction of reoperations that is more than twice as high as the increase in overall mastectomies.

**Key Points:**

• *Confounding factors cause imbalance when investigating the influence of preoperative MRI on surgical outcomes of pure DCIS.*

• *When patient matching is applied to women with pure unilateral DCIS, reoperation rates are significantly reduced in women who underwent preoperative MRI.*

• *The reduction of reoperations brought about by preoperative MRI is more than double the increase in overall mastectomies.*

## Introduction

The role of preoperative breast magnetic resonance imaging (MRI) in guiding the treatment of ductal carcinoma in situ (DCIS) diagnosed at core needle biopsy (CNB) or vacuum-assisted biopsy (VAB) is an open issue in clinical practice [1–4] that has been extensively investigated considering different outcomes, e.g., short-term surgical results [5–16], upgrade to invasive cancer at final pathology [17, 18], detection of additional ipsilateral or contralateral disease [19], long-term recurrence [20], and patient preferences [21, 22]. Surgical outcomes are crucial, as the rate of mastectomy and the rate of reoperation after breast-conserving surgery are major indicators of breast care quality and the focus of multidisciplinary efforts towards surgical de-escalation [23–25].

As shown by three systematic reviews published between 2015 and 2021 [26–28], cohort studies that investigated the effects of preoperative MRI on surgical outcomes of DCIS generally exhibit a referral bias towards MRI for young patients with extensive and high-grade DCIS. These characteristics represent strong confounding factors, being intrinsically associated with poor outcomes and ultimately prompting more aggressive surgery [27, 29]. Naturally, these studies outline contrasting scenarios and add to ongoing uncertainty about the impact of preoperative MRI on surgical outcomes [27, 28].

However, different results emerge from the very few studies adopting methods to minimize or remove the referral bias [30]. Save for the very small subgroups of DCIS in the MONET randomized controlled trial [31], as of mid-2023, only two studies present such characteristics: the IRCIS randomized controlled trial [11] and the propensity score matching study by Yoon et al [15]. Pooled together, data from these two studies show that, compared to patients not referred to preoperative MRI, patients with DCIS undergoing preoperative MRI have a 1.52 odds ratio (OR) of undergoing mastectomy as their initial surgery, a 1.89 OR of having negative margins and avoiding reoperation, and a 0.97 OR of overall mastectomy (at first-line surgery or at reoperation). Nonetheless, as noted in the conclusions of a systematic review by the European Commission Initiative on Breast Cancer working groups [27], estimates from these studies remain affected by low statistical power.

In this context, the present study takes advantage of the large cohort of patients with DCIS registered in the database of the Multicenter International Prospective Analysis (MIPA) study [32–34], which allows the application of methods for confounder adjustment while still retaining a large number of patients. Thus, we aimed to compare in matched cohorts the surgical outcomes of patients with DCIS referred or not referred to preoperative MRI, namely the first-line mastectomy rate, the reoperation rate, and the overall mastectomy rate.

## Materials and methods

### Study design

This is a subgroup analysis of data from the MIPA study (ISRCTN41143178), whose design is detailed in the protocol paper [32]. In summary, MIPA is a prospective observational study conducted in 27 centers worldwide between June 2013 and November 2018, after approval from the Ethics Committee of the coordinating center (Comitato Etico Ospedale San Raffaele, Milano, Italy; protocol number 2784). Each center consecutively enrolled women aged 18–80 years with newly diagnosed breast cancer, without indication for neoadjuvant therapy and amenable to upfront surgery. In accordance with the observational nature of the study, each multidisciplinary team followed local routine practice in the diagnostic and therapeutic pathway, including the decision on whether to refer patients to bilateral contrast-enhanced preoperative MRI after conventional imaging with digital mammography and/or ultrasonography (US).

### Study population and endpoints

According to the aforementioned aims, this study focuses on patients with a diagnosis of pure unilateral DCIS at CNB/VAB, as the presence of ipsilateral and/or contralateral invasive cancer and the presence of bilateral DCIS are known to influence surgical management [35, 36], acting as strong potential confounding factors.

Following the study protocol [32], surgical endpoints for all patients in this analysis are (i) first-line mastectomy; (ii) immediate/short-term reoperation for close or positive margins; and (iii) overall mastectomy (i.e., performance of mastectomy as first-line surgery or at reoperation). Due to the focus on unilateral lesions, the secondary endpoint of first-line bilateral mastectomy is excluded from this analysis.

Conversely, data on non-surgical secondary study endpoints based on surgical pathology (such as the upgrade of DCIS to invasive cancer and complete DCIS removal) will not be considered in this analysis and will be separately reported for all patients with pure DCIS at CNB/VAB.

### Patient matching

The nonrandomized observational design of the MIPA study implies the existence of different selection biases towards the referral to MRI, yielding a skewed distribution of several characteristics (i.e., covariates) between patients who underwent MRI before surgery (MRI group) and those who did not undergo MRI (noMRI group). Thus, patient matching was implemented to reduce covariate imbalance, to estimate the average effect of preoperative MRI on surgical outcomes in the population at clinical equipoise, i.e., patients with overlapping baseline characteristics [37, 38].

To strictly adhere to the real-world workflow of breast cancer care, the following baseline characteristics—all already available before the decision to refer (or not) patients to MRI—were considered covariates for patient matching (Table 1): age; hormonal status; presence of familial breast cancer risk^1^; posterior-to-nipple diameter^2^; highest BI-RADS category at conventional imaging; lesion diameter at conventional imaging; lesion presentation at conventional imaging^3^; surgical planning after conventional imaging.

**Table 1:** Comparison of baseline demographic, clinical, and imaging characteristics in the unmatched and matched cohorts

		Unmatched cohorts				Matched cohorts			
		noMRI	MRI	*p*	SMD	noMRI	MRI	*p*	SMD
Patients		498	507	–	–	309	309	–	–
Mean age (SD)		59 years (10)	56 years (10)	< 0.001	0.267	57 years (10)	57 years (9)	0.865	0.013
Hormonal status	*Premenopausal*	118 (23.8%)	140 (27.6%)	0.229	0.132	85 (27.5%)	75 (24.3%)	0.462	0.022
	*Perimenopausal*	49 (9.9%)	62 (12.2%)			32 (10.4%)	43 (13.9%)		
	*Receiving HRT*	3 (0.6%)	2 (0.4%)			2 (0.6%)	1 (0.3%)		
	*Post-menopausal*	326 (65.7%)	303 (59.8%)			190 (61.5%)	190 (61.5%)		
Patients with familial breast cancer risk		5 (1.0%)	11 (2.2%)	0.224	0.093	1 (0.3%)	1 (0.3%)	1.000	< 0.001
Mean posterior-to-nipple diameter (SD)		96.2 mm (31.9)	89.6 mm (30.3)	0.001	0.213	92.2 mm (26.5)	90.9 mm (27.9)	0.554	0.043
Highest BI-RADS at conventional imaging	*BI-RADS 0*	5 (1.0%)	10 (2.0%)	< 0.001	0.354	1 (0.3%)	1 (0.3%)	1.000	< 0.001
	*BI-RADS 1*	0 (0.0%)	4 (0.8%)			0 (0.0%)	0 (0.0%)		
	*BI-RADS 2*	0 (0.0%)	8 (1.6%)			0 (0.0%)	0 (0.0%)		
	*BI-RADS 3*	14 (2.8%)	33 (6.5%)			7 (2.3%)	7 (2.3%)		
	*BI-RADS 4*	363 (72.9%)	319 (63.0%)			238 (77.0%)	238 (77.0%)		
	*BI-RADS 5*	116 (23.3%)	132 (26.1%)			63 (20.4%)	63 (20.4%)		
Lesion presentation at conventional imaging	*Unifocal*	433 (86.9%)	425 (83.8%)	0.014	0.185	286 (92.6%)	286 (92.6%)	1.000	< 0.001
	*Multifocal*	55 (11.0%)	54 (10.7%)			21 (6.8%)	21 (6.8%)		
	*Multicentric*	10 (2.0%)	28 (5.5%)			2 (0.6%)	2 (0.6%)		
Mean lesion diameter at conventional imaging (SD)		20.9 mm (20.4)	24.0 mm (20.3)	0.022	0.151	18.0 mm (14.0)	18.3 mm (13.4)	0.790	0.014
Planned mastectomy after conventional imaging		67 (13.5%)	125 (24.7%)	< 0.001	0.288	31 (10.0%)	31 (10.0%)	1.000	< 0.001

*SMD* standardized mean difference, *SD* standard deviation, *HRT* hormone replacement therapy

Using the “MatchIt” package [39] on R (version 4.2.1, The R Foundation for Statistical Computing), nearest neighbor 1:1 matching with the rank-based robust Mahalanobis distance [40, 41] was performed with specifications chosen to optimize covariate balance by taking advantage of the large number of patients in the MIPA study: (i) exact 1:1 matching was enforced for the following categorical covariates: familial breast cancer risk, highest BI-RADS at conventional imaging, lesion presentation at conventional imaging, and surgical planning after conventional imaging; (ii) calipers were applied in the matching of the following continuous covariates: age (caliper width: ± 0.5 standard deviations), posterior-to-nipple diameter (caliper width: ± 2 standard deviations), maximum lesion diameter at conventional imaging (caliper width: ± 0.5 standard deviations). Matching was performed without replacement, and unmatched patients were discarded.

Covariate balance between the MRI and noMRI group was assessed before and after matching by calculating the standardized mean difference for all variables and by performing two-tailed Pearson’s *χ*^2^ or Fisher’s exact tests for categorical variables and the Mann–Whitney *U* test for continuous variables. In order to consider matching successful, a conservative combined balance threshold was applied, with all covariates having to display standardized mean differences ≤ 0.050 with *p* values ≥ 0.100 [42].

### Comparison of surgical endpoints

Comparisons of the three surgical endpoints in the unmatched and matched cohorts were carried out with two-tailed Pearson’s *χ*^2^ or Fisher’s exact tests, as appropriate, after calculation of the respective ORs. To account for multiple testing, the Bonferroni correction was applied considering the 6 comparisons of surgical endpoints in the unmatched and matched cohorts, with an ensuing *p* < 0.008 threshold for statistical significance. All analyses were performed with R (version 4.2.1, The R Foundation for Statistical Computing) and STATA (version MP 17.1, StataCorp).

## Results

### Study population

**Fig. 1:**
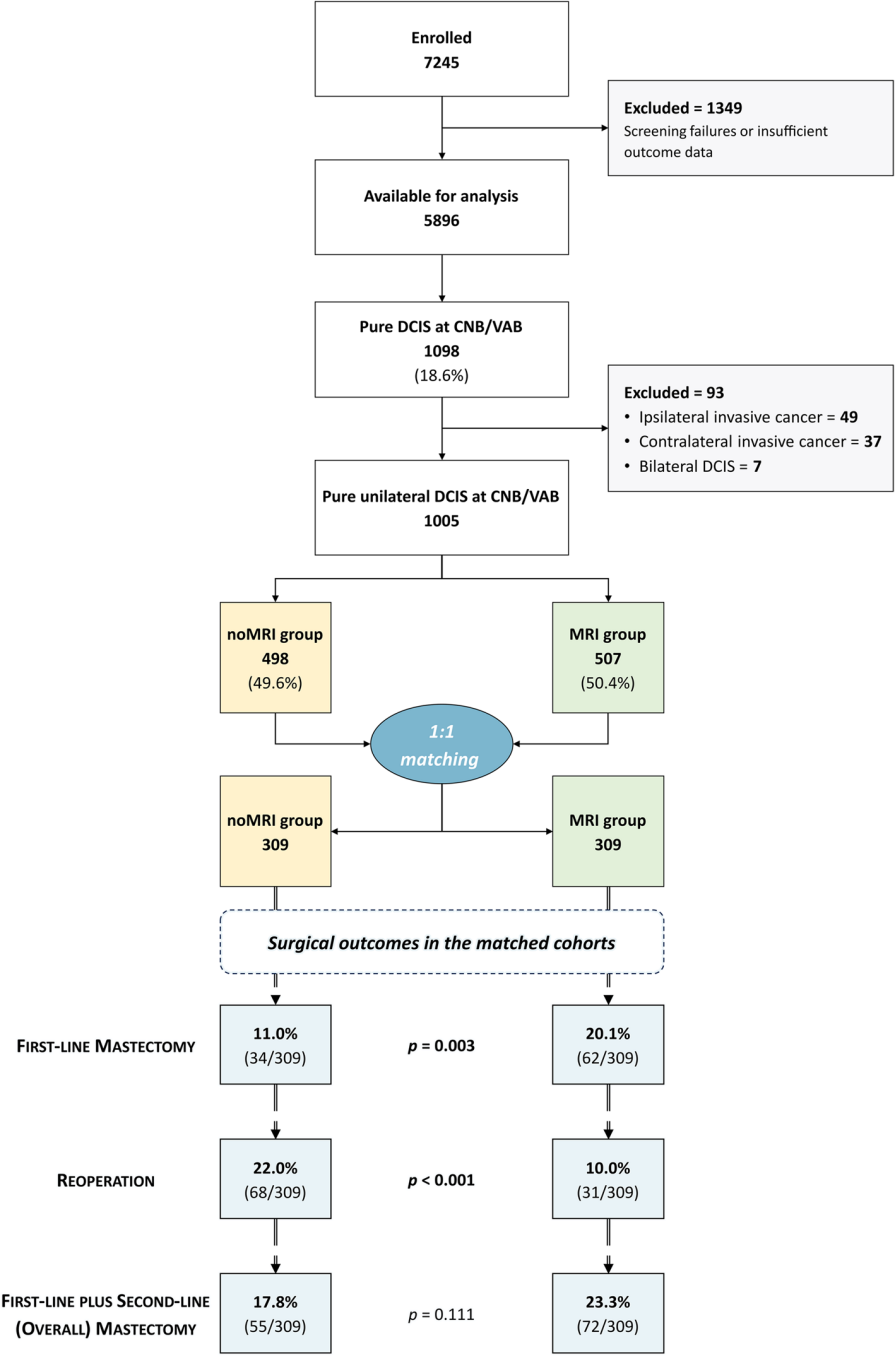
Study flowchart and surgical outcomes in the matched cohorts

As described in the study flowchart (Fig. 1), 5896 among the 7245 patients enrolled between June 2013 and November 2018 had sufficient information to be considered for this analysis. At least one lesion diagnosed as pure DCIS at CNB/VAB was present in 1098/5896 patients (18.6%): applying the exclusion criteria, 37/1098 (3.4%) patients were excluded because of the presence of a contralateral invasive cancer, 49/1098 (4.5%) because of the presence of another ipsilateral lesion diagnosed as invasive cancer, and 7/1098 (0.6%) because of the presence of bilateral pure DCIS. Thus, 1005 patients with pure unilateral DCIS at CNB/VAB were included in this analysis, 507/1005 (50.4%) in the MRI group and 498/1005 (49.6%) in the noMRI group.

As detailed in Table 2, tissue sampling was most frequently prompted by mammographic findings (884/1005 patients, 87.9%), either alone (540/1005 patients, 53.7%) or in combination with US or MRI (344/1005 patients, 34.2%). A total of 658/1005 (65.5%) samplings were performed with VAB, while the remaining 347/1005 (34.5%) with CNB: accordingly, stereotaxis was the most frequent biopsy guidance (673/1005 patients, 67.0%), followed by US (310/1005 patients, 30.8%) and MRI (22/1005 patients, 2.2%).

**Table 2:** Modality of detection of the findings prompting tissue sampling

Tissue sampling prompt	Number	%
Mammography alone	540/1005	53.7%
US alone	93/1005	9.3%
MRI alone	20/1005	2.0%
Mammography + US	194/1005	19.3%
Mammography + MRI	89/1005	8.8%
US + MRI	8/1005	0.8%
Mammography + US + MRI	61/1005	6.1%

*US* ultrasonography, *MRI* magnetic resonance imaging

### Unmatched cohorts—baseline characteristics

Before matching, different distributions between the noMRI and the MRI group were observed for six of the eight baseline descriptors (Table 1). Patients in the MRI group were younger than those in the noMRI group (mean age 56 years versus 59 years, *p* < 0.001) and also differed in DCIS presentation at conventional imaging, displaying larger lesions (mean diameter 24.0 versus 20.9 mm, *p* = 0.022) that were more frequently multifocal or multicentric (16.2% versus 13.0%, *p* = 0.014). Finally, patients in the MRI group had a + 11.2% difference in the referral to mastectomy after conventional imaging compared to patients in the noMRI group (125/507 patients in the MRI group, 24.7%, versus 67/498 patients in the noMRI group, 13.5%).

### Unmatched cohorts—surgical endpoints

As detailed in Table 3, the + 11.2% difference in the referral to mastectomy in the MRI group after conventional imaging rose to + 19.2% in the evaluation of first-line surgery: specifically, the first-line mastectomy rate was 33.5% in the MRI group (170/507 patients) compared to 14.3% (71/498 patients) in the noMRI group (OR 3.03, *p* < 0.001). While the 12.4% (63/498 patients) reoperation rate of the MRI group was significantly lower (− 7.7%, OR 0.56, *p* = 0.001) than the 20.1% reoperation rate of the noMRI group (100/498 patients), the overall rate of mastectomy in the MRI group (190/507 patients, 37.5%) was still 17.2% higher (OR 2.36, *p* < 0.001) than that of the noMRI group (101/498 patients, 20.3%).

**Table 3:** Comparison of surgical outcomes in the unmatched and matched cohorts

	Unmatched cohorts 1005 patients					Matched cohorts 618 patients				
	noMRI group 498 patients	MRI group 507 patients	Difference for the MRI group			noMRI group 309 patients	MRI group 309 patients	Difference for the MRI group		
			%	OR	*p*			%	OR	*p*
First-line mastectomy	71 (14.3%)	170 (33.5%)	+ 19.2%	3.03	< 0.001	34 (11.0%)	62 (20.1%)	+ 9.1%	2.03	0.003
Reoperation	100 (20.1%)	63 (12.4%)	− 7.7%	0.56	0.001	68 (22.0%)	31 (10.0%)	− 12.0%	0.40	< 0.001
Overall mastectomy (first-line + second-line)	101 (20.3%)	190 (37.5%)	+ 17.2%	2.36	< 0.001	55 (17.8%)	72 (23.3%)	+ 5.5%	1.40	0.111

*MRI* magnetic resonance imaging, *OR* odds ratio

### Matched cohorts—baseline characteristics

A total of 618 patients were matched, 309 in each group. As detailed in Table 1, matched patients had a median age of 57 years, being mostly post-menopausal (380/618, 61.5%). At conventional imaging, the most frequent BI-RADS classifications were BI-RADS 4 in 476/618 patients (77.0%) and BI-RADS 5 in 126/618 patients (20.4%). The vast majority of matched patients (572/618, 92.6%) had unifocal presentation at conventional imaging, 42/618 (6.8%) having multifocal DCIS, and only 4/618 patients (0.6%) presenting with multicentric DCIS.

### Matched cohorts—surgical endpoints

Starting from the matched 10.0% rate of referral to mastectomy at conventional imaging (31/309 patients in both groups; Table 3), the MRI group still had a significantly higher first-line mastectomy rate (20.1%, 62/309 patients, OR 2.03) compared to the noMRI group (11.0%, 34/309 patients, *p* = 0.003). However, the reoperation rate in the MRI group (10.0%, 31/309 patients, OR for reoperation 0.40) was less than half that of the noMRI group (22.0%, 68/309 patients, *p* < 0.001), corresponding to a 2.53 OR of avoiding reoperation for women in the MRI group. This resulted in a non-significant difference (*p* = 0.111) in the overall mastectomy rate for the MRI group (23.3%, 72/309 patients, OR 1.40) compared to the noMRI group (17.8%, 55/309 patients).

## Discussion

This subgroup analysis of the MIPA study focused on 1005 patients with pure unilateral DCIS at CNB/VAB who underwent (507 patients, 50.4%) or did not undergo (498 patients, 49.6%) preoperative MRI, evaluating differences in surgical outcomes between the MRI and noMRI groups. After 1:1 patient matching according to eight covariates concerning demographic, clinical, and imaging characteristics, 309 patients were matched in each group; the significantly higher first-line mastectomy rate of the MRI group (20.1% versus 11.0% in the noMRI group) was counterbalanced by an even higher decrease of reoperations (10.0% in the MRI group versus 22.0% in the noMRI group), culminating in a 5.5% increase in the overall mastectomy rate for the MRI group (23.3% vs 17.8% in the noMRI group) that will need to be clinically contextualized with follow-up data.

As already mentioned, the interpretation of our results must consider methodological peculiarities and limitations both of this study and of previous ones. In the unmatched cohorts, data from all three surgical outcomes (first-line mastectomy, reoperation, overall mastectomy) are in line with pooled data of previous cohort studies reported by the working groups of the European Commission Initiative on Breast Cancer [27]: in our study—before patient matching—preoperative MRI led patients with pure DCIS at CNB/VAB to an even lower OR for reoperation (0.56 versus a pooled 0.72) but to higher OR for first-line mastectomy (3.03 versus a pooled 2.04) and overall mastectomy (2.36 versus a pooled 1.58).

**Fig. 2:**
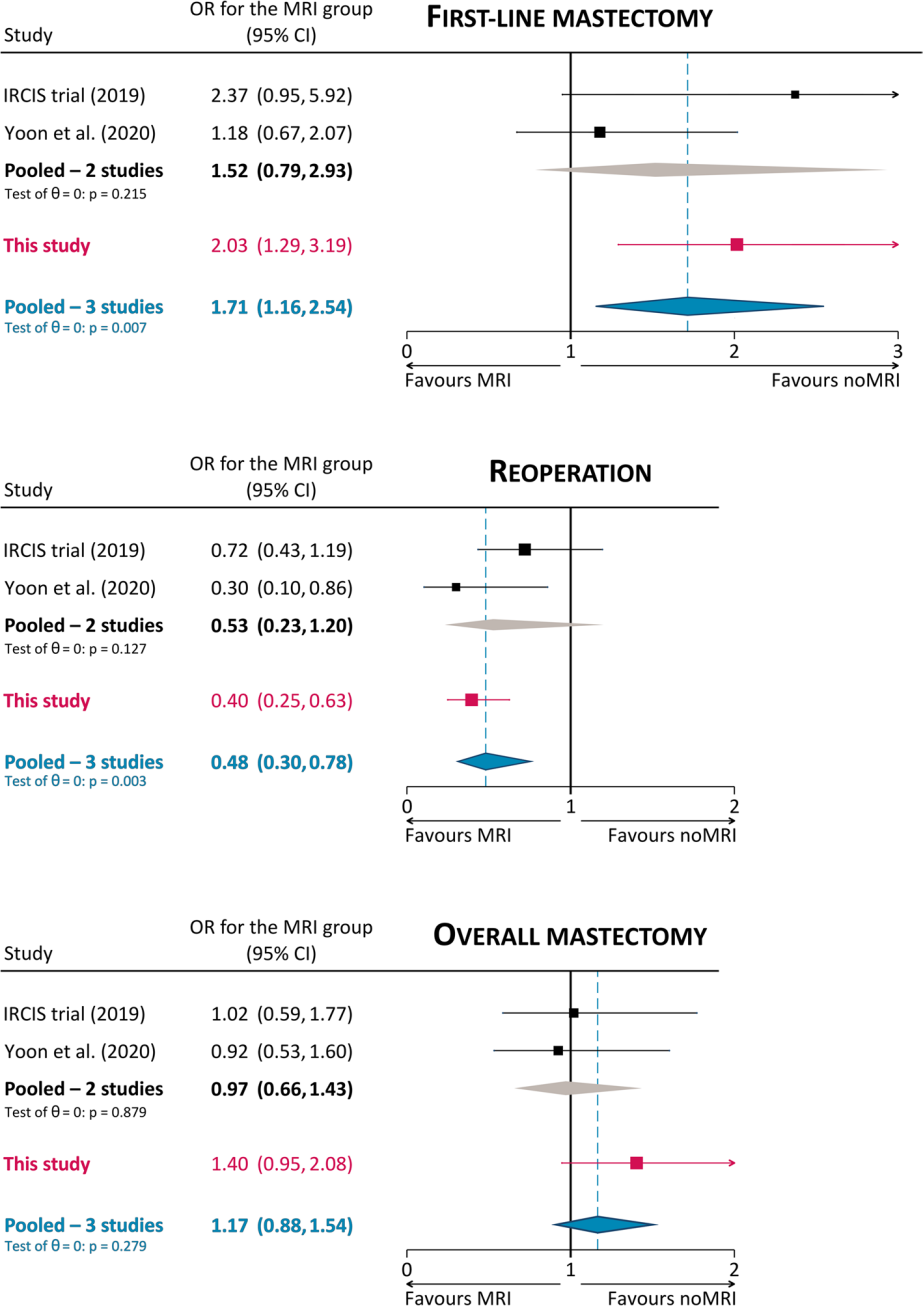
Explorative comparison of surgical outcomes with previous controlled analyses. Odds ratios (OR) refer to women with DCIS undergoing preoperative MRI, with the noMRI group as the reference

Importantly, in the matched cohort, data from our study confirmed the results obtained by previous studies where randomization [11] or propensity score matching [15] were implemented to deal with confounding factors. As shown in Fig. 2, the 2.03 OR for first-line mastectomy of women in the MRI group was higher than the 1.18 OR obtained by Yoon et al [15] but lower than the 2.37 OR in the IRCIS trial [11]: of note, adding our data to the pooling, the ensuing 1.71 pooled OR reached statistical significance (*p* = 0.007), substantiating the association between MRI and first-line mastectomy. However, a similar finding could be observed for the protective effect of MRI towards reoperation, underlined by the 0.40 OR in our study, slightly higher than the 0.30 OR obtained by Yoon et al [15] but almost half of the 0.72 OR in the IRCIS trial [11]: adding our data to the pooling confirmed the protective effect of MRI for reoperation, with a significant 0.48 pooled OR (*p* = 0.003). For the last endpoint (i.e., overall mastectomy), the 1.40 OR of MRI found in our study was higher than the protective 0.92 OR found by Yoon et al [15] and the 1.02 OR found in the IRCIS trial [11]. Nonetheless, as in these two studies, the confidence interval of this OR crosses the no-effect line, not reaching statistical significance (*p* = 0.111, pooled OR 1.71 with *p* = 0.279). Notably, our overall mastectomy rates in the matched cohorts (23.3% in the MRI group, 17.8% in the noMRI group) were about half of those found by Yoon et al (38.7% in the MRI group, 40.6% in the noMRI group) [15] and closely comparable to those of the IRCIS trial (17.6% in the MRI group, 17.3% in the noMRI group) [11].

At an overall appraisal, results from our study emphasize that when CNB/VAB yields a DCIS diagnosis, women undergoing preoperative MRI have a 2.03 OR of receiving mastectomy as their first-line surgery that is counterbalanced by the 2.53 OR of avoiding reoperation. The focus on biopsy data represents a strong point of our study and allows for the translation of these results into clinical practice, as multidisciplinary teams (and surgeons in particular) do not know in advance the final pathology report—where the case under discussion would be confirmed as a pure DCIS or a DCIS associated with an invasive cancer—but base their decision-making on CNB/VAB results and on what is suggested by imaging findings, in particular extent of calcifications on mammograms, hypoechoic findings at US, or enhancement at MRI. Finally, the non-significant 1.40 OR of the MRI group for overall mastectomy—also due to the loss of cases from the matching process—leaves open the question on whether this OR and the absolute percentage differences found for this surgical outcome (5.5% in our study, 1.9% and 0.3% in the other two studies [11, 15]) are clinically relevant (also in a long-term perspective that will be explored with follow-up data) and justify the conduction of studies adequately powered to detect significant differences in this endpoint, as highlighted by a cost-effectiveness analysis conducted on data from the IRCIS trial [43].

The limitations of this work can be ascribed to two macro-areas, i.e., general limitations of the MIPA study itself and limitations specifically pertaining this subgroup analysis. As for the general limitations of the MIPA study, its nonrandomized observational design remains the chief obstacle to a controlled evaluation of surgical outcomes. However, we addressed this issue with the aid of patient matching according to a large number of covariates, also employing conservative matching methods with strict criteria. While this represents a potential solution to avoid some issues of randomized controlled trials such as high costs, statistical power issues, and poor external validity and representativeness of results obtained in highly controlled settings [44, 45], we acknowledge that our analysis could not account for several other patient-specific or institutional potentially confounding factors. Of these, the most difficult to model remains the effect of the surgical habits at each institution and even of each surgeon in a given institution [46–50]. Another general limitation of the MIPA study is its wide enrolment timeframe, during which the quick expansion of the role of digital breast tomosynthesis and of MRI itself in the diagnostic setting could have created hidden imbalances between and inside subgroups.

The main specific limitation of this targeted analysis on needle biopsy–diagnosed DCIS is the fact that the MIPA study database did not collect information on DCIS grade and receptor status at CNB/VAB, acquiring these data only from surgical pathology. Had it been available, DCIS grade at CNB/VAB would have represented a covariate for matching, considering not only its prognostic implications [51] but also its specific influence on the accuracy of MRI [52] and on the interplay between preoperative MRI and surgical outcomes [10, 12, 16]. Again referring to the 2013–2018 enrolment timeframe of the MIPA study, this subgroup analysis could not account for the impact of the progressive clinical introduction of 3-T MRI systems, which are known to improve DCIS differential diagnosis and the accuracy of lesion sizing [53–55], nor for the potential competition in the preoperative setting between MRI and other contrast-enhanced imaging modalities, such as contrast-enhanced mammography [56–58].

In conclusion, this subgroup analysis of the MIPA study showed that, when surgical outcomes of women diagnosed with pure DCIS at CNB/VAB are compared in matched cohorts, the increase in the overall mastectomy rate engendered by preoperative MRI is less than half the corresponding reduction in reoperation rates.

## Abbreviations

CNB: Core needle biopsy
DCIS: Ductal carcinoma in situ
MIPA: Multicenter International Prospective Analysis
MRI: Magnetic resonance imaging
OR: Odds ratio
US: Ultrasonography
VAB: Vacuum-assisted biopsy

## Footnotes

1 Defined as the presence of 3 or more first-degree relatives with breast or ovarian cancer.

2 Measured on the craniocaudal mammographic view from the posterior image limit to the basis of the nipple.

3 Unifocal; multifocal; multicentric.

## Declarations

### Guarantor

The scientific guarantor of this publication is Prof. Francesco Sardanelli, MD.

### Statistics and biometry

The first three authors (A.C., G.D.L, N.H.) have significant statistical expertise.

### Study subjects or cohorts overlap

This paper is a targeted subanalysis of 1005 patients that were previously included in the main paper of the MIPA study (Sardanelli et al, European Radiology 2022; 10.1007/s00330-021-08240-x) and in the paper comparing patients with different MRI referrals (Cozzi et al, European Radiology 2023; 10.1007/s00330-023-09600-5).

### Methodology

• prospective

• observational

• multicenter study
